# Features of HTLV-1 transcriptional and chromatin remodeling dynamics emerge from ChIP-chip analysis

**DOI:** 10.1186/1742-4690-8-S1-A181

**Published:** 2011-06-06

**Authors:** Mohammad Heydarian, Dowser Alani, Irene Guendel, Rachel Van Duyne, Tim McCaffrey, Sidney W Fu, Kylene Kehn-Hall, Fatah Kashanchi

**Affiliations:** 1 John Hopkins University, Department of Biological Chemistry, Baltimore, MD, 21205, USA; 2 George Mason University, Department of Molecular and Microbiology, National Center for Biodefense and Infectious Diseases, Manassas, VA, 20110, USA; 3 The George Washington University Medical Center, Department of Microbiology, Immunology, and Tropical Medicine, Washington, DC, 20037, USA; 4 The George Washington University Medical Center, Department of Medicine, Washington, DC, 20037, USA

## 

Human T-lymphotropic virus type 1 (HTLV-1) is the etiological agent of adult T cell leukemia (ATL), a lymphoproliferative disease that primarily affects CD4+ T cells. Cellular transformation of infected cells is mediated through Tax, a multifaceted viral-encoded transactivator protein, which exerts regulatory effects in gene expression and cell cycle dysregulation. Vast differential cellular gene expression changes have been documented in the presence of Tax. We have performed ChIP-chip experiments and confirmation of gene expression dysregulation in uninfected and infected cell lines and performed analysis on selected microarray data from published literature (Tesi cells). We have characterized the resulting changes into seven categories according to the absence or presence of transcriptional machinery and core chromatin remodeling complex components RNA polymerase II (Pol II), CREB, BRG1, NFκB, and Tax, on the HTLV-1 long terminal repeat (LTR). Our data set suggests that gene expression regulation upon infection results in activation, de-repression, suppression (elongation block), suppression (block in factor occupancy), suppression (initiation block) and possible Pol II-independent gene expression in the presence of Tax. We have previously shown the essential Tax-BRG1 interaction for Tax transactivation and viral production, and that additional recruitment of transcriptional machinery and chromatin remodeling complexes to the viral LTR results in differential gene modulation.

